# Supplementary material for: Resting Energy Expenditure in the Elderly: Systematic Review and Comparison of Equations in an Experimental Population
Source: Nutrients. 2021 Jan 29;13(2):458. doi: 10.3390/nu13020458 (PMC7912404; doi:10.3390/nu13020458)
Supplement: Supplementary file 1 [file nutrients-13-00458-s001.pdf]

Online Supplementary Materials - Ocagli et al.

Table S1. Search strategy in Medline, Scopus and Embase.

**Search terms**
**Medline**
((("Energy Intake"[MeSH] OR "Energy Intake" [Text Word]) OR ("energy intake/physiology" [Text Word]) OR ("Basal Metabolism" [Text Word]) OR "basal metabolism"[MeSH] OR basal metabolic rate[Text Word] OR ("Nutritional Requirements" [MeSH]) OR ("Nutritional Requirements" [Text Word]) OR ("Nutritional Requirement" [Text Word]) OR ("Resting metabolic rate" [Text Word]) OR ("Resting energy expenditure" [Text Word]) OR ("Energy expenditure" [Text Word]) OR (“Metabolism” [MeSH]) OR (“energy metabolism" [Text Word]) OR (“energy metabolism"[MeSH] OR "energy metabolism"[MeSH] OR energy expenditure[Text Word]))
**AND**
(“predictive equation*” OR “prediction equation*”)
**Scopus**
("Energy Intake” OR "energy intake/physiology" OR "Basal Metabolism*" OR “basal metabolic rate” OR “Basal Energy Expenditure” OR "Nutritional Requirement*" OR "Resting metabolic rate*" OR "Resting energy expenditure*" OR "Energy expenditure*” OR “Metabolism” OR “energy metabolism" OR “energy expenditure”)
**AND**
((“predictive equations” [Text Word]) OR (“predictive equation” [Text Word]) OR (“prediction equations” [Text Word]) OR (“prediction equation” [Text Word])))
**Embase**
('caloric intake'/exp OR 'basal metabolic rate'/exp OR 'nutritional requirement*' OR 'resting metabolic rate'/exp OR 'resting energy expenditure*' OR 'metabolism'/exp OR 'energy metabolism'/exp OR 'energy expenditure*'/exp OR 'estimated energy expenditure' OR 'estimated energy requirement')
**AND**
('equation*' OR 'predictive equation*')

Table S2. Variables reported in retrieved equations grouped in homogeneous categories. For each variable is reported the frequency of utilization in the equations both in equations validated in elderly and young population.

Variables		N° predictive equations		
		Elderly	Young	Total
**Demographic characteristics**	Age	26	121	147
	Gender	33	133	166
	Ethnicity	6	2	8
	Menopausal Status	0	1	1
	Smoke	0	6	6
	Meal	0	1	1
**Meaurements**	Height	19	67	86
	Weight	31	152	183
	BMI*	4	24	28
	Abdomen Circumference	0	1	1
	Hip Circumference	0	4	4
	Waist Circumference	2	1	3
	Wrist Circunference	0	2	2
	Mid arm Circumference	0	4	4
	Subscapular Skinfold	0	1	1
	Arm span	0	3	3
	Chest Skinfold	0	1	1
**Measure of fat percentage**	Lean Body Mass	0	2	2
	Surface Area	2	4	6
**Clinical Condition**	Diabetes	0	2	2
	NYHA** class	0	1	1
**Laboratory Tests**	Albumin	0	1	1
	Glycemia	0	2	1
	CPR***	0	1	1
**Physical Activity**	Physical Activity	0	6	6
	Athletics	0	3	3
	Leisure Time Activity	0	1	1
**Enviromental Measure**	Temperature	2	2	4
	Humidity	0	1	1
	Time	0	1	1
**Vital Parameters**	Body Temperature	0	1	1
	Heart rate	0	1	1
	Blood pressure	0	4	4

*BMI = Body Mass Index, **NYHA = New York Heart Association ***, CPR = C reactive protein

Table S3. Estimated BMR, RMR and EEE for each equation according to gender in the patients of the nursing home. Variables considered in the structure of the equations and I, II, and III quartile are showed for each equation.

Equation	Variables considered	N	Female (N=27)	Male (N=60)	Combined (N=87)
Aleman [35]	W, G	86	1305/1396/1531	1055/1181/1284	1144/1266/1375
Anjos [36]	G, W, H, A	73	1027/1121/1250	820/ 969/1052	911/1018/1131
Arciero [37]	G, W, H, MS	48	1061/1194/1249		1061/1194/1249
Bernstein [39]	G, W, H, A	73	736/ 857/1015	893/ 983/1050	848/ 963/1049
Bernstein [39]	G, A, BSA	73	1570/1713/1899	779/ 919/ 973	858/ 974/1570
Camps [40]	G, W	86	1407/1491/1616	1078/1194/1288	1160/1286/1429
Carrasco [41]	G, A ≥ 30, W	86	1413/1488/1599	1121/1222/1304	1192/1303/1430
Carrasco [41]	G, W, 18<A<74	86	1314/1409/1519	1006/1116/1206	1082/1197/1326
Cunningham [43]	LBM	86	1425/1520/1615	1169/1272/1336	1244/1327/1427
Cunningham [43]	LBM, A	86	1295/1376/1489	1037/1140/1201	1099/1188/1296
De la Cruz [88]	G, W, A	86	1391/1467/1627	937/1063/1153	1013/1151/1381
De Lorenzo [89]	G, W, H, A	73	1187/1306/1484	942/1124/1196	1036/1177/1306
De Luis [90]	G, W, H, A	73	1231/1327/1505	1050/1186/1261	1145/1231/1334
EU [44]	G, A>75, W	86	1313/1369/1451	1099/1189/1263	1163/1257/1347
EU [44]	G, W, 60<A<74	86	1403/1483/1601	1135/1221/1290	1196/1288/1410
Frankenfield [45]	G, W, H, A	86	1222/1326/1488	1132/1832/1954	1148/1398/1888
Frankenfield [45]	G, W, H, A	86	1222/1326/1488	1132/1832/1954	1148/1398/1888
Frankenfield [46]	G, A, W, H	73	1042/1100/1208	939/1013/1042	966/1023/1100
Fredrix [47]	G, W, A	86	1346/1419/1593	1001/1126/1212	1079/1205/1358
Freni [48]	G, W	86	1400/1487/1616	1125/1210/1279	1185/1277/1404
Freni [48]	G, W, A	86	1120/1210/1368	1031/1126/1203	1062/1150/1227
Gaillard [49]	G, W	73	1123/1260/1432	931/1159/1264	1036/1200/1336
Gaillard [49]	W, H, A	73	1157/1259/1403	928/1119/1191	1043/1157/1277
Gaillard [49]	W	86	1180/1258/1373	1059/1166/1253	1096/1216/1305
Ganpule [50]	G, W, H, A	73	1314/1418/1568	962/1147/1231	1075/1230/1394
Harris & Benedict [52]	G, H, W, A	73	1109/1236/1436	972/1116/1193	1037/1159/1240
Henry [54]	G, A>60, W	86	1309/1400/1533	1058/1152/1228	1124/1222/1336
Henry [54]	G, A>70, W	86	1333/1420/1548	1066/1161/1237	1133/1231/1353
Henry [54]	G, 60<A<70, W	86	1288/1380/1515	1062/1154/1229	1127/1222/1317
Huang [55]	G, D, A, W, H	73	1398/1525/1651	1010/1116/1186	1071/1192/1398
Ikeda [56]	G, W, A	86	1220/1307/1408	986/1085/1167	1051/1154/1260

IOM [57]	G, PL, A, W, H (BMI)	73	1834/2086/2235	1325/1476/1628	1398/1638/1907
IOM [57]	G, A, W, H (NW)	54	1264/1328/1456	987/1091/1193	1036/1194/1293
IOM [57]	G, A, W; H	73	1298/1388/1525	999/1139/1202	1084/1202/1325
IOM [57]	G, PL, A, W, H (BMI)	49	1906/2129/2247	1456/1586/1666	1568/1708/2086
Kashiwazaki [58]	W, SS	59	1469/1665/1826	1243/1477/1641	1351/1547/1716
Korth [59]	G, W, A, H	73	1365/1470/1609	855/1052/1114	980/1131/1370
Korth [59]	W	86	1469/1574/1730	1306/1451/1568	1355/1518/1638
Kruizenga [60]	G, BMI, W, H	73	1414/1527/1683	1029/1231/1309	1168/1307/1478
Lam [61]	G, W, H, A	73	1641/1749/1900	1172/1364/1443	1281/1448/1660
Lazzer [62]	F: W, H; M: W, H, A	73	1155/1279/1436	1074/1231/1325	1119/1248/1380
Lazzer [62]	G, W, A	86	1425/1521/1631	1033/1142/1232	1104/1225/1421
Leung [63]	W, A	86	1096/1196/1356	925/1093/1178	988/1114/1224
Liu [64]	G, W, H, A	73	596/734/859	547/715/827	565/719/836
Liu [64]	W	86	1224/1360/1561	1012/1200/1352	1076/1287/1443
Liu [64]	W, H	73	1149/1278/1476	843/1052/1181	998/1120/1293
Liu [64]	G, W, A	86	1307/1435/1582	994/1144/1265	1097/1244/1374
Livingston [65]	G, A, BMI	86	1238/1329/1456	913/1049/1116	991/1107/1250
Livingston [65]	G, A	86	1384/1456/1556	1262/1371/1452	1300/1418/1498
Livingston [65]	G, W	86	1083/1176/1305	920/1070/1155	983/1094/1187
Livingston [65]	W	86	1521/1596/1700	1174/1273/1346	1245/1345/1516
Luhrmann [66]	W	86	1231/1337/1494	1065/1212/1331	1115/1280/1402
Luhrmann [66]	G, W, A	86	1342/1446/1567	1033/1152/1250	1110/1239/1382
Luhrmann [66]	G, W	86	1391/1473/1593	1085/1197/1288	1164/1286/1415
Lv [67]	G, A, H, W, PL	43	545/ 829/1172	316/ 504/ 729	382/ 654/ 954
Metsios [68]	A, W, CRP	17	1317/1399/1458	1041/1213/1331	1067/1275/1431
Mifflin [69]	G, W, H, A	73	1166/1268/1410	774/ 962/1030	914/1045/1208
Mifflin [69]	W	86	1260/1362/1511	1103/1242/1356	1150/1307/1423
Mifflin [69]	G, W	86	1428/1511/1633	1114/1215/1297	1186/1296/1436
Moore [70]	G, W	86	1629/1706/1820	1198/1329/1434	1290/1433/1632
Muller [71]	G, W, A	86	1388/1486/1599	1024/1135/1227	1096/1220/1389
Muller [71]	G, W, A, H	73	1374/1432/1509	1020/1135/1166	1094/1173/1374
Muller [71]	G, W, A, BMI	47	1485/1559/1651	1130/1194/1217	1165/1213/1447
Obisesan [72]	HF, W, PG, Al	13	1442/1578/1604	1294/1359/1442	1312/1426/1578
Orozco [93]	G, E, BMI, W, A	45	1386/1449/1607	1008/1088/1152	1068/1150/1360
Owen [73]	G, PL, W	80	1478/1541/1636	1143/1209/1262	1190/1262/1473

Pavlidou [74]	BMI	73	919/1030/1176	747/ 934/1010	824/ 964/1074
Pavlidou [74]	BMI, G	73	757/846/958	569/708/766	657/749/846
Quenoille [75]	H, W, BSA, AT, h	71	1339/1432/1532	1194/1320/1387	1263/1348/1450
Roza [94]	G, H, W, A	73	1167/1291/1477	952/1107/1174	1035/1158/1278
Roza [94]	G, H, W, A	73	1111/1238/1437	970/1115/1195	1037/1160/1242
Roza [94]	G, BMI, A	73	1432/1576/1741	1442/1613/1831	1436/1609/1811
Sabounchi [21]	W, H	73	1135/1225/1359	960/1121/1186	1039/1156/1252
Schofield [77]	G, W	86	1277/1356/1472	1099/1183/1251	1158/1239/1329
Schofield [77]	G, W, H	73	1191/1277/1372	996/1133/1230	1086/1183/1298
Segura [78]	G, W, H, A	73	1006/1093/1202	661/ 878/ 962	834/ 955/1093
Segura [78]	G, W, H	73	1151/1199/1279	785/ 974/1071	922/1082/1189
Segura [78]	G, W, H, BMI, A	73	893/1017/1280	706/ 815/ 894	787/ 872/ 979
Segura [78]	G, BMI, W, H, A	62	1443/1790/2325	619/ 818/ 897	716/ 888/1126
Siervo [95]	G, BMI, W	31		1249/1291/1350	1249/1291/1350
Silver [79]	W	86	1237/1378/1586	1017/1212/1369	1084/1302/1463
Silver [79]	W	86	1296/1443/1661	1066/1269/1434	1135/1364/1533
Silver [79]	W	86	1355/1509/1736	1114/1327/1500	1187/1426/1603
Soares [96]	W, A	86	1271/1373/1489	1145/1259/1357	1197/1297/1386
Tabata [82]		87	1284/1284/1398	1014/1014/1014	1014/1014/1284
Tabata [82]	G, BMI, A	27	1058/1182/1355		1058/1182/1355
Tabata [83]	G, BMI, A	73	1177/1269/1431	895/1020/1202	962/1173/1286
Tabata [83]	G, W, A	27	1058/1182/1355		1058/1182/1355
Valencia [97]	G, W	86	879/ 974/1115	1052/1154/1236	997/1124/1227
Vander Weg [98]	G, W, H, A	73	1093/1173/1289	912/1061/1118	998/1097/1173
Weijs & Vansant [84]	G, BMI, W, H, A	45	1516/1624/1712	1200/1281/1353	1250/1344/1513
Wilms [86]	A, W	86	1187/1284/1395	1066/1175/1266	1115/1209/1295
Who [85]	G, W, H	73	1203/1293/1429	1041/1188/1267	1123/1214/1335
Wright [99]	G, W, A, H	73	1183/1276/1420	730/ 913/ 972	871/ 977/1184
Wright [99]	G, BMI, W, H, A	45	1327/1374/1546	795/ 947/1048	837/1041/1313
Yang [100]	G, W	86	1463/1605/1816	1097/1294/1453	1236/1425/1573
Yang [100]	G, W	86	1464/1632/1881	1123/1275/1399	1223/1380/1541
Yang [100]	BSA	74	1173/1231/1373	818/ 930/1045	865/1042/1191
Yang [100]	W	86	1413/1596/1866	1129/1381/1585	1214/1498/1707
Yangmei [101]	G, A, AT, Wac, PL	73	1538/1664/1875	1191/1462/1733	1316/1548/1750

Abbreviations: G = gender, W = weight, H = height, A = Age, MS = Menopausal Status, D = diabetic, E = ethnicity, CRP = C reactive protein, HF = heart failure, PG = plasma glucose, Al = albumin, PL = physical level, BSA = body

surface area, h = humidity, AT = Ambiental temperature, BMI = body mass index, NW = normal weight, SS = subscapular skinfold, Wac = waist circumference.

Figure S1. Forest plots reporting the Intraclass Correlation Coefficient (ICC) with 95% CI of estimated REE according to the category of patients that were not considered in predictive equations for specific groups of predictive equations. The vertical grey line represents the ICC in each category without any grouping.

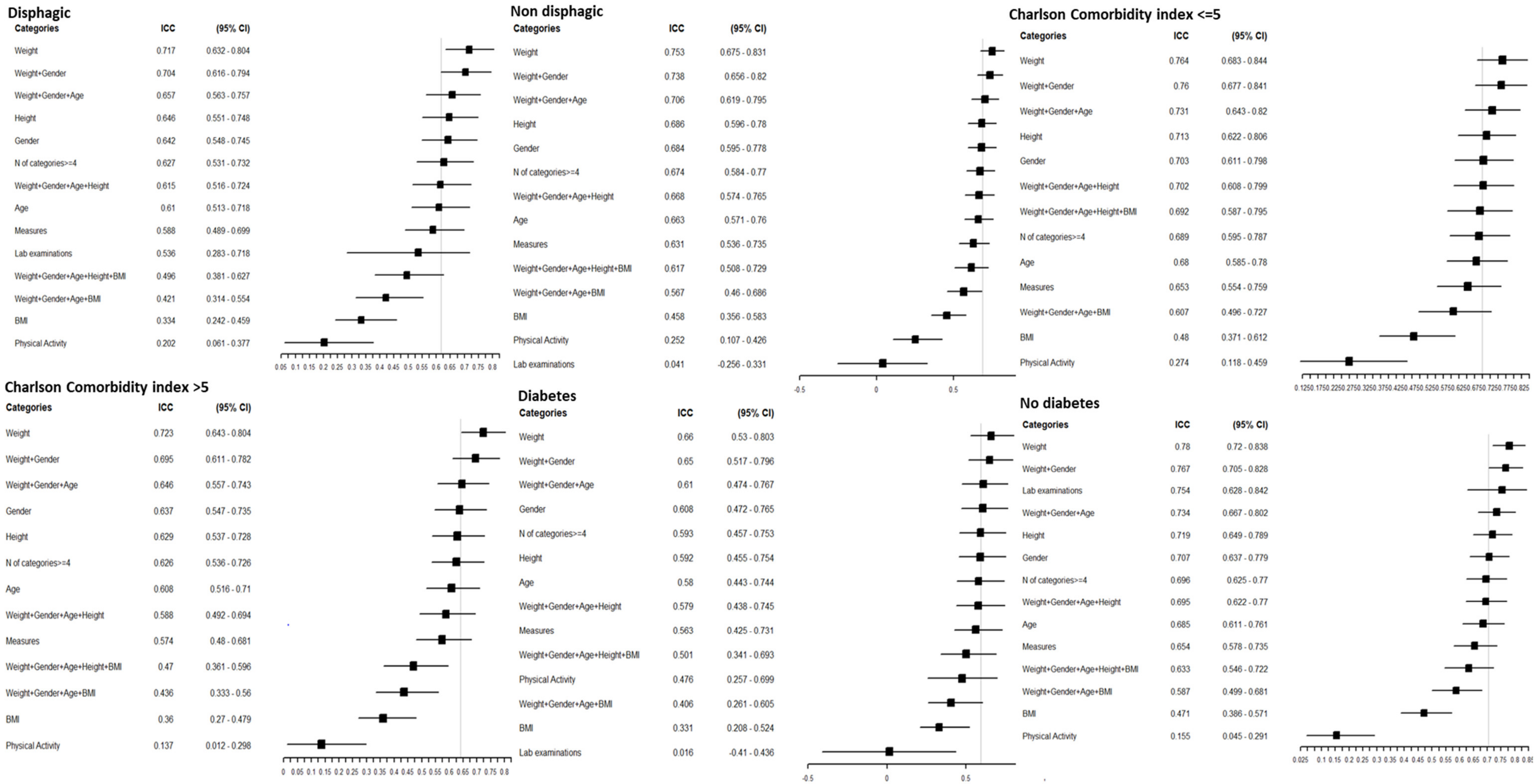

Figure S2. Agreement among predictive equations in terms of Kcal/die at individual level among the categories gender, BMI, Charlson Comorbidity Index, presence/absence of dysphagia and diabetes. For each category is reported the median estimated REE at 95% CI considering all the predictive equations.

Individual Categories	Mean intake	(95% CI)
Male	1411	(1319-1503)
Obese	1364	(1255-1473)
Charlson<5	1286	(1222-1350)
Dysphagia no	1280	(1216-1344)
Diabetes yes	1240	(1153-1327)
Diabetes no	1228	(1177-1279)
Normal weight	1195	(1137-1253)
Charlson>=5	1179	(1120-1238)
Dysphagia yes	1173	(1115-1231)
Female	1145	(1098-1192)

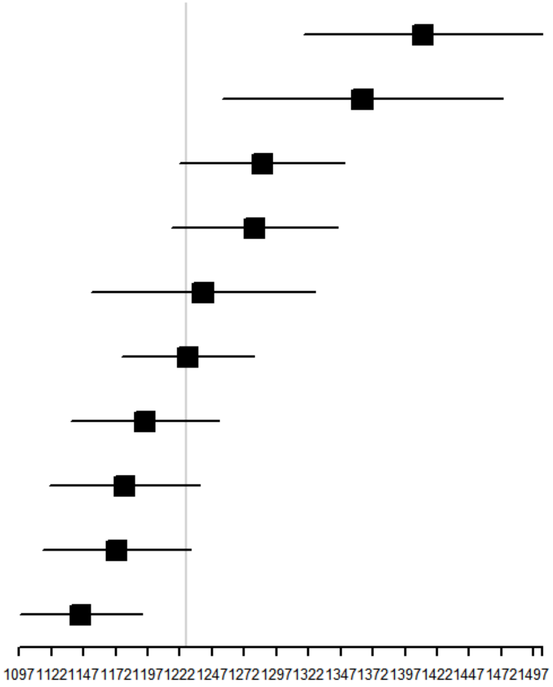

Figure S3. ICC among BMR, RMR and EEE with 95% CI for all predictive equations.

Categories	ICC	95% IC
BEE-BMR	0.796	0.745-0.846
REE-RMR	0.756	0.701-0.812
EEE-EER	0.457	0.364-0.558
overall	0.68	0.62-0.75

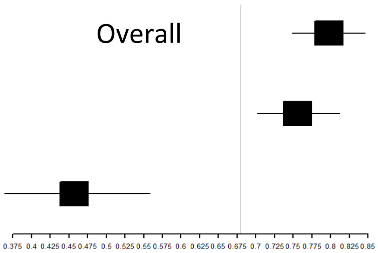

Categories	ICC	95% IC
BEE-BMR	0.804	0.714-0.886
REE-RMR	0.677	0.563-0.799
EEE-EER	0.254	0.113-0.451
overall	0.64	0.52-0.77

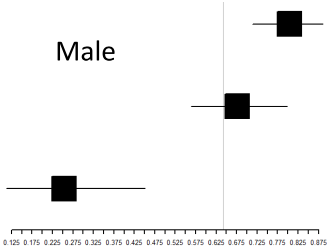

Categories	ICC	95% IC
REE-RMR	0.871	0.829-0.91
BEE-BMR	0.791	0.729-0.851
EEE-EER	0.1	0.024-0.205
overall	0.67	0.59-0.75

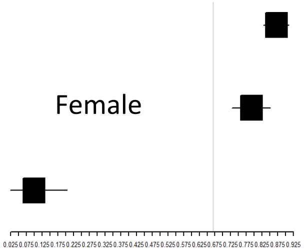

Categories	ICC	95% IC
REE-RMR	0.86	0.777-0.931
BEE-BMR	0.828	0.728-0.914
EEE-EER	0.587	0.4-0.775
overall	0.82	0.72-0.9

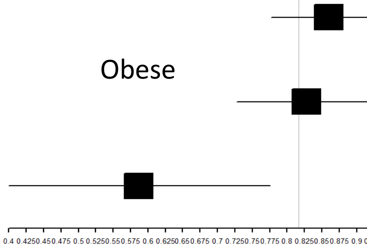

Categories	ICC	95% IC
BEE-BMR	0.803	0.747-0.856
REE-RMR	0.713	0.644-0.784
EEE-EER	0.585	0.486-0.685
overall	0.67	0.59-0.74

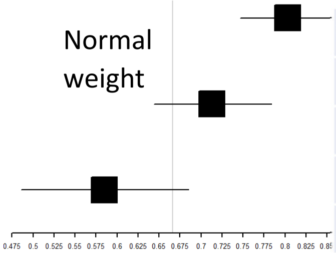

Categories	ICC	95% IC
BEE-BMR	0.763	0.683-0.84
REE-RMR	0.692	0.602-0.785
EEE-EER	0.361	0.253-0.532
overall	0.52	0.62-0.72

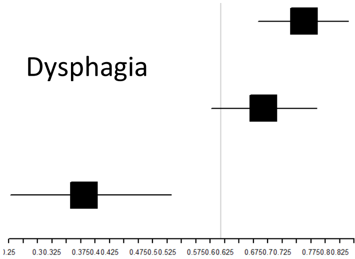

Categories	ICC	95% IC
BEE-BMR	0.806	0.736-0.87
REE-RMR	0.771	0.696-0.845
EEE-EER	0.479	0.35-0.62
overall	0.69	0.61-0.78

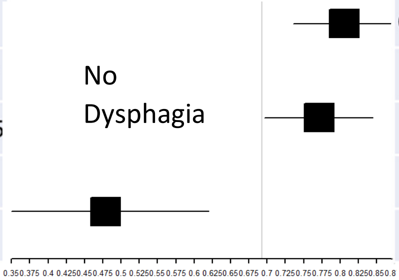

Categories	ICC	95% IC
BEE-BMR	0.806	0.736-0.87
REE-RMR	0.771	0.696-0.845
EEE-EER	0.479	0.35-0.62
overall	0.69	0.61-0.78

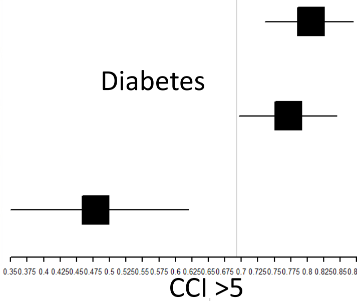

Categories	ICC	95% IC
BEE-BMR	0.808	0.752-0.86
REE-RMR	0.795	0.738-0.85
EEE-EER	0.415	0.309-0.534
overall	0.70	0.63-0.78

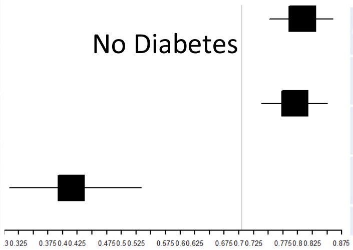

Categories	ICC	95% IC
BEE-BMR	0.789	0.719-0.856
REE-RMR	0.712	0.63-0.796
EEE-EER	0.389	0.268-0.532
overall	0.64	0.55-0.63

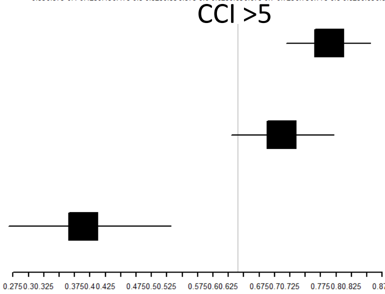

Categories	ICC	95% IC
BEE-BMR	0.789	0.719-0.856
REE-RMR	0.712	0.63-0.796
EEE-EER	0.389	0.268-0.532
overall	0.70	0.61-0.80

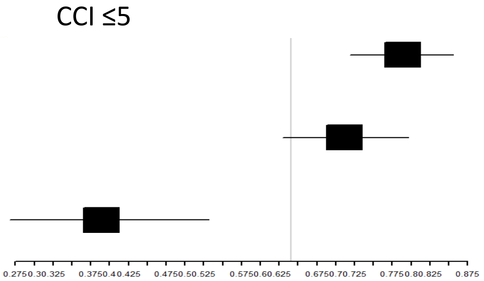

Figure S4. Example of plots visualized in the shiny app Equationer in a female of 65 kg and 75 years at first as BMR, in the second plot as RMR and in the third with information on physical activity. The graphics in red included the information on gender, in grey are equations that do not distinguish between males and females.

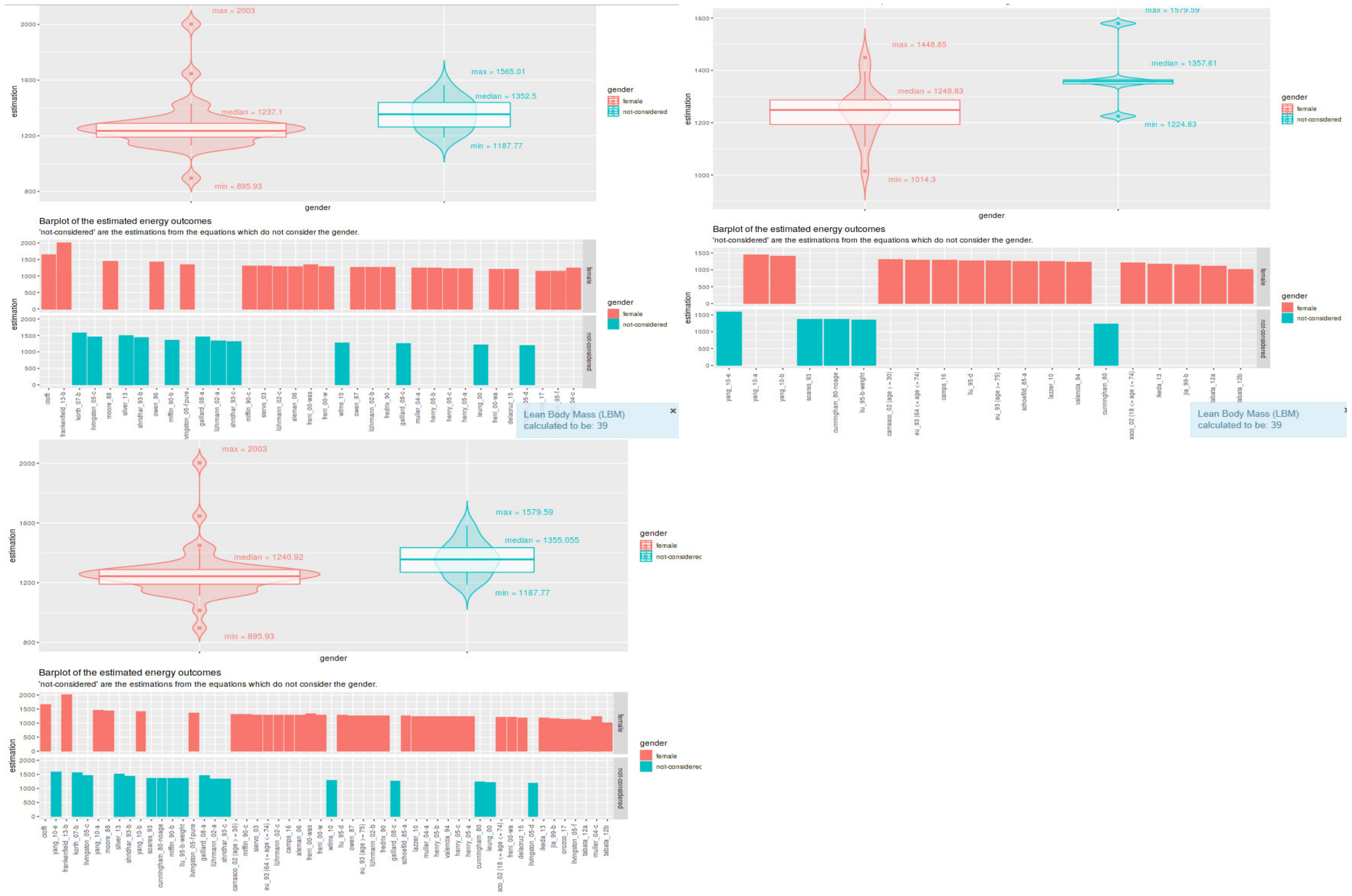

Text S1. Instructions for the use of the web-application

To estimate energy requirements in elderly patients, we created an R Shiny app available at the following link https://r-ubesp.dctv.unipd.it/shiny/equationer/. The interface is designed to let the user insert the characteristics available of the patients, then the app computes the outcome according to the equations retrieved in this review. Results will be shown both graphically and in a tabular manner in order to be more comprehensive. The patient's information will not be memorized, and the connexion is secured according to SSL 256 standards. The interface is based on an R package called "equationer" available at https://github.com/UBESP-DCTV/equationer.

Example of usage:

We have data of a male patient that has 85 years old, weight 75 kg, and is diabetic.

1) At first, we select the variable age, and weight imputing the data available, as shown in Figure 1. In this page are shown other sections that collects different kind of variables.

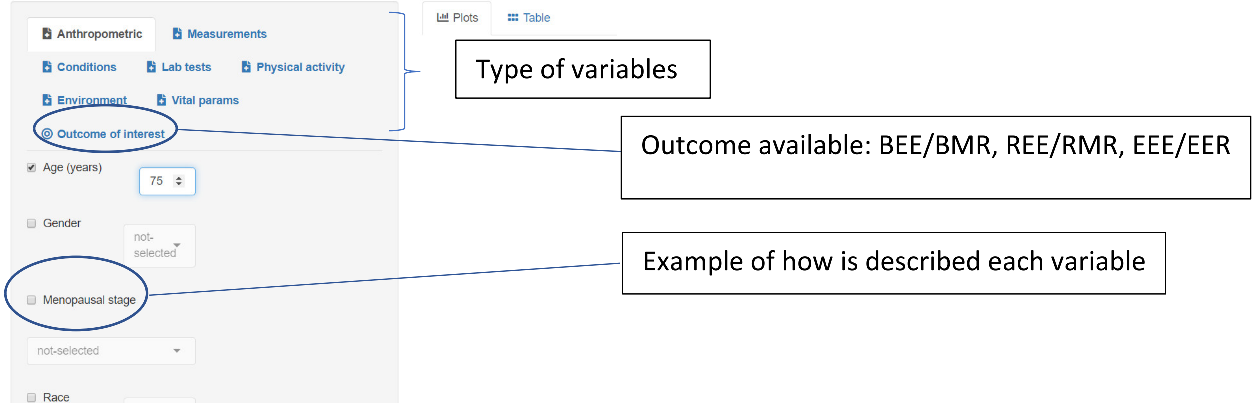

Figure 1. Example of app visualization of the imputation of the "covariates."

Some variables will be evaluated automatically, such as Body Mass index, ideal body weight, and lean body mass according to the equations provided in the studies (Figure 2).

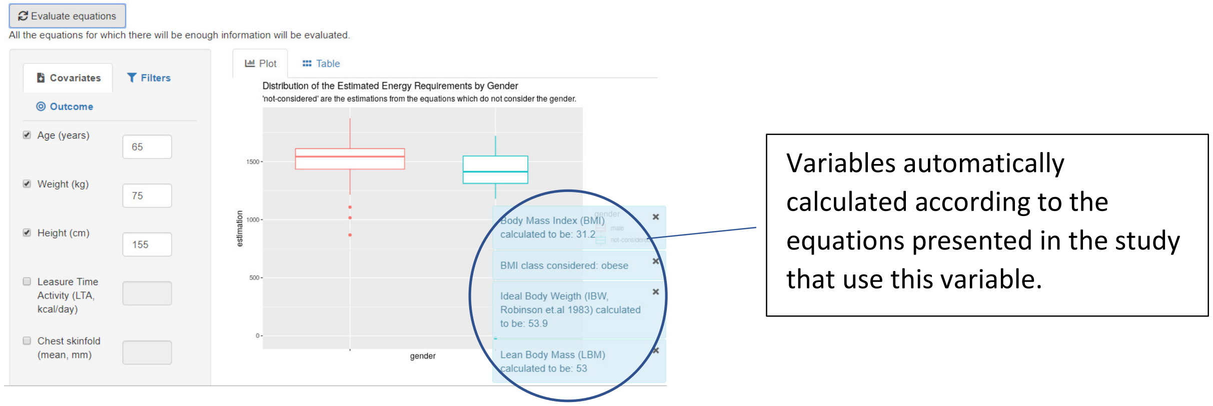

Figure 2. Example of variables calculated automatically

2) Then we can choose the outcome, which is the definition of the caloric intake, like RMR, or BEE (Figure 3). The names are the ones used in the study validation. Outcomes are automatically selected, but if you wish to consider only equations with a specific definition of the outcome, it is possible to uncheck the other ones. The more outcomes remain selected, the more results will be displayed.

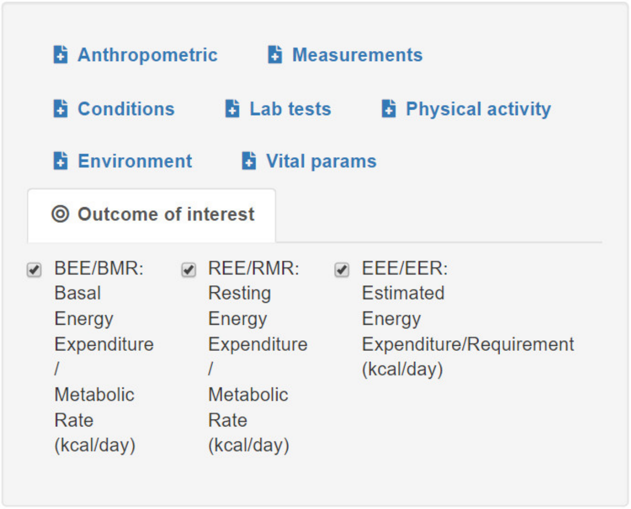

Figure 3. Outcome considered in the app

3) At last, clicking on the 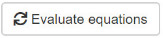

button, you can visualize the results in graphical manner clicking on “plot” (Figure 4) both in boxplot form and barplot.

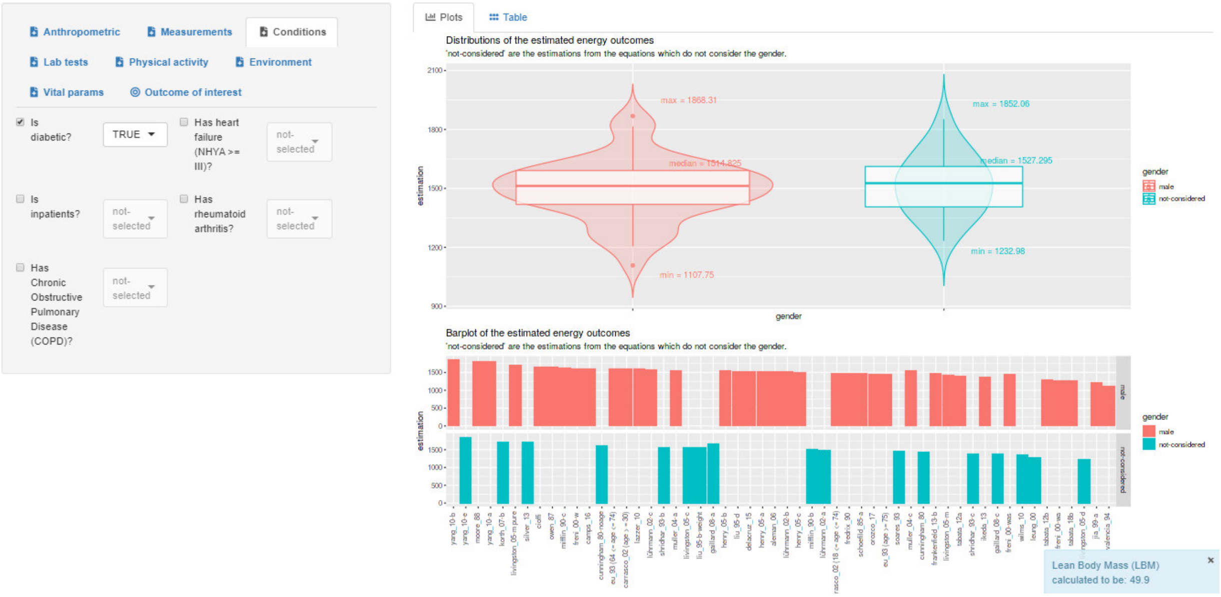

Figure 4. Example of graphical visualization in the app

Results are available also in tabular manner, as shown in Figure 5. The tabular view of the results shows all the equation used for the calculation of the outcome in the patients presented before. The table also presents the variables presented in each equation in order to guide the physician in the choice of the proper equation for his patients.

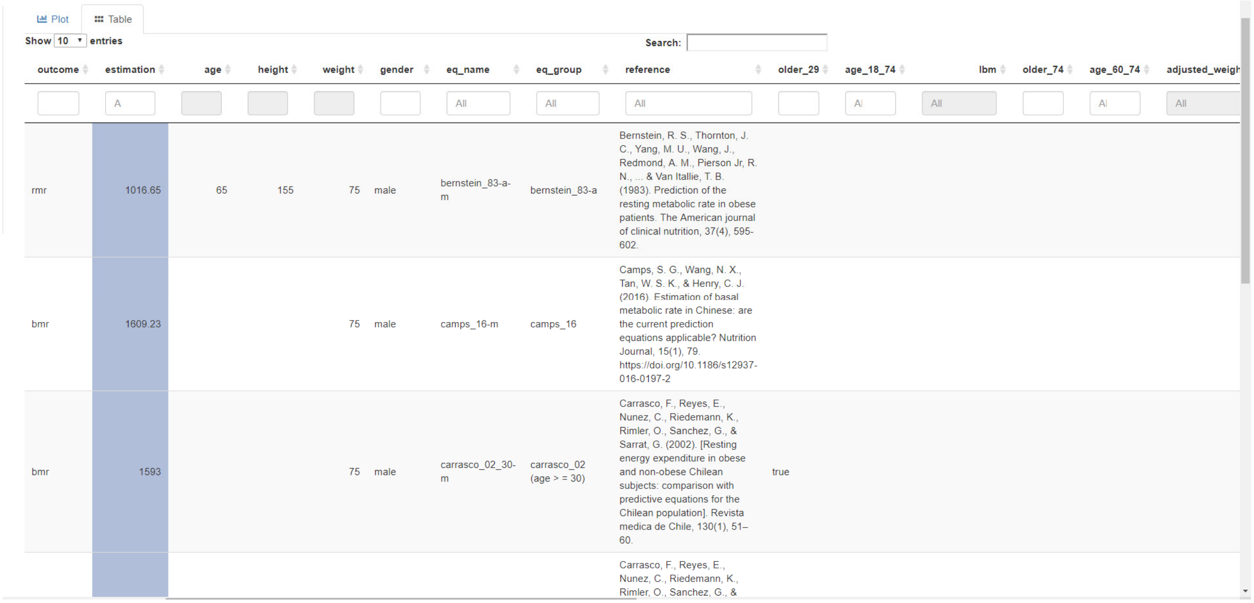

Plot Table

Show 10 entries

Search:

outcome	estimation	age	height	weight	gender	eq_name	eq_group	reference	older_29	age_18_74	lbm	older_74	age_60_74	adjusted_weigh
	A					All	All	All		Al	All		Al	All
rmr	1016.65	65	155	75	male	bernstein_83-a-m	bernstein_83-a	Bernstein, R. S., Thornton, J. C., Yang, M. U., Wang, J., Redmond, A. M., Pierson Jr, R. N., ... & Van Itallie, T. B. (1983). Prediction of the resting metabolic rate in obese patients. The American journal of clinical nutrition, 37(4), 595-602.						
bmr	1609.23			75	male	camps_16-m	camps_16	Camps, S. G., Wang, N. X., Tan, W. S. K., & Henry, C. J. (2016). Estimation of basal metabolic rate in Chinese: are the current prediction equations applicable? Nutrition Journal, 15(1), 79. https://doi.org/10.1186/s12937-016-0197-2						
bmr	1593			75	male	carrasco_02_30-m	carrasco_02 (age > = 30)	Carrasco, F., Reyes, E., Nunez, C., Riedemann, K., Rimler, O., Sanchez, G., & Sarrat, G. (2002). [Resting energy expenditure in obese and non-obese Chilean subjects: comparison with predictive equations for the Chilean population]. Revista medica de Chile, 130(1), 51–60.	true					
								Carrasco, F., Reyes, E., Nunez, C., Riedemann, K., Rimler, O., Sanchez, G., &						

Figure 5. Example of tabular results
